# Adherence to a lifestyle monitoring system in patients with heart disease: protocol for the care-on prospective observational trial

**DOI:** 10.1186/s12872-023-03222-x

**Published:** 2023-04-17

**Authors:** W. F. Goevaerts, N. C. C. W. Tenbült - van Limpt, W. J. Kop, M. V. Birk, Y. Liu, R. W. M. Brouwers, Y. Lu, H. M. C. Kemps

**Affiliations:** 1 Industrial Design, Eindhoven University of Technology, Eindhoven, The Netherlands; 2 Department of Cardiology, Máxima Medical Centre, Eindhoven/Veldhoven, The Netherlands; 3 Department of Medical and Clinical Psychology, Center of Research on Psychological Disorders and Somatic Diseases, Tilburg University, Tilburg, the Netherlands

**Keywords:** Cardiac rehabilitation, Continuous monitoring, Lifestyle, Physical activity, Stress management, Dietary behavior management, Sleep monitoring, Adherence, Usability, Acceptance

## Abstract

**Background:**

Lifestyle factors such as physical fitness, dietary habits, mental stress, and sleep quality, are strong predictors of the occurrence, clinical course, and overall treatment outcomes of common cardiovascular diseases. However, these lifestyle factors are rarely monitored, nor used in daily clinical practice and personalized cardiac care. Moreover, non-adherence to long-term self-reporting of these lifestyle factors is common. In the present study, we evaluate adherence to a continuous unobtrusive and patient-friendly lifestyle monitoring system using evidence-based assessment tools.

**Methods:**

In a prospective observational trial (*N* = 100), the project investigates usability of and adherence to a monitoring system for multiple lifestyle factors relevant to cardiovascular disease, i.e., daily physical activity levels, dietary habits, mental stress, smoking, and sleep quality. Patients with coronary artery disease, valvular disease and arrhythmias undergoing an elective intervention are asked to participate. The monitoring system consists of a secured online platform with a custom-built conversational interface—a chatbot—and a wrist-worn wearable medical device. The wrist-worn device collects continuous objective data on physical activity and the chatbot is used to collect self-report data. Participants collect self-reported lifestyle data via the chatbot for a maximum of 4 days every other week; in the same week physiological data are collected for 7 days for 24 h. Data collection starts one week before the intervention and continues until 1-year after discharge. Via a dashboard, patients can observe their lifestyle measures and adherence to self-reporting, set and track personal goals, and share their lifestyle data with practitioners and relatives. The primary outcome of the trial is adherence to using the integrated platform for self-tracking data. The secondary outcomes include system usability, determinants of adherence and the relation between baseline lifestyle behaviour and long-term patient-relevant outcomes.

**Discussion:**

Systematic monitoring during daily life is essential to gain insights into patients’ lifestyle behaviour. In this context, adherence to monitoring systems is critical for cardiologists and other care providers to monitor recovery after a cardiac intervention and to detect clinical deterioration. With this project, we will evaluate patients’ adherence to lifestyle monitoring technology. This work contributes to the understanding of patient-centered data collection and interpretation, to enable personalized care after cardiac interventions in order to ultimately improve patient-relevant outcomes and reduce health care costs.

**Trial registration:**

Netherlands Trial Registry (NTR) NL9861. Registered 6th of November 2021.

## Background

Optimization of lifestyle behavior and psychological wellbeing are considered pivotal in cardiac rehabilitation, as physical fitness [1], daily physical activity (PA) levels [2], dietary habits [2], mental stress [2], sleep quality [3, 4], and smoking habits [5] are strongly related to the occurrence, clinical course and overall treatment results of common cardiovascular diseases (CVD’s) such as coronary artery disease (CAD) and cardiac arrhythmias such as atrial fibrillation (AF) [6]. Moreover, quality of life is often not improved after major cardiac interventions [7–9], which may be due to adverse effects of persistent unhealthy lifestyle behaviour on the clinical course of coronary artery disease and atrial fibrillation [9]. However, despite its undisputed relevance, lifestyle behaviour is currently not monitored systematically and therefore not optimally used to the advantage of patients in daily practice [8, 10].

In order to successfully implement lifestyle monitoring in cardiac care pathways, optimizing adherence to self-tracking of lifestyle behaviour via monitoring technologies is essential. Without data provided by the patients, personalized and improved treatment decisions cannot be made. However, there is a gap in literature regarding the adherence to continuous lifestyle monitoring technologies for a longer period of time. Whereas previous research showed high levels of adherence associated with monitoring technology [11, 12], these studies focused on relatively short programs (mostly 1 to 12 weeks). Secondly, studies typically focus on monitoring of one rather than multiple lifestyle domains. Yet, the use of technology to monitor multiple lifestyle domains over a prolonged period of time may be particularly useful as an assistive tool to achieve a healthy lifestyle, and subsequently, better health outcomes in special populations [11]. Therefore, there is a clear need for further research in evaluation of the adherence and usability of this kind of digital health technology in CVD care and management [13].

The Care-On trial evaluates patients’ adherence to monitoring of lifestyle behaviours (i.e. daily physical activity levels, dietary habits, mental stress and sleep quality) with a newly designed system that integrates innovative methods for continuous unobtrusive and patient-friendly in patients with coronary artery disease, valvular disease and arrhythmias. Subsequent system usability, determinants of adherence and the relation between baseline lifestyle behaviour and long-term patient-relevant outcomes are investigated. We postulate that a system that aids patients in monitoring their lifestyle factors will enable better self-management and improve self-motivation [14], with subsequent positive effects on the lifestyle factors themselves and, eventually, on long-term clinical outcomes.

## Methods

### Study design

**Fig. 1:**
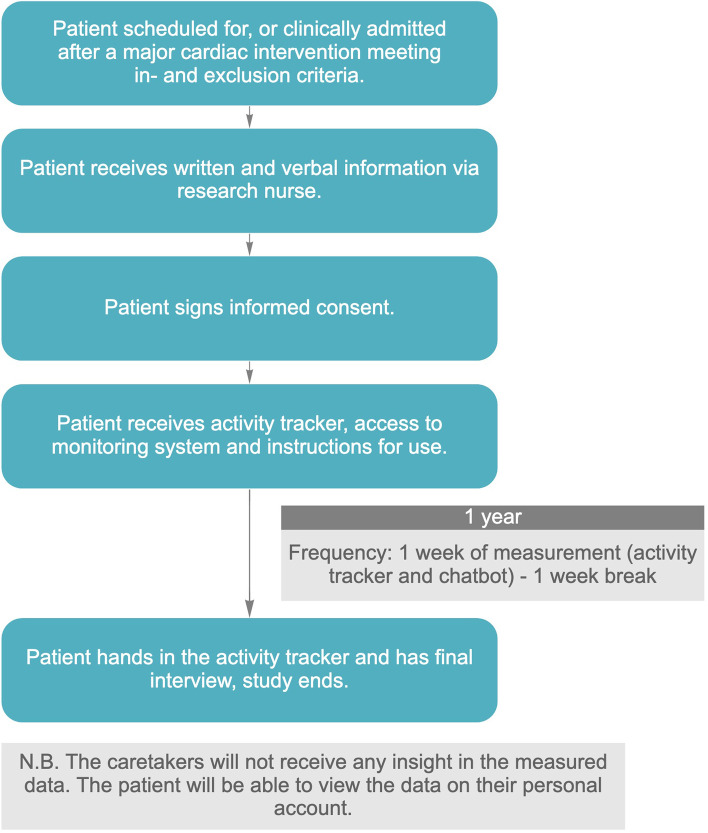
Study design of the Care-On prospective observational trial

This study is designed as a monocenter prospective observational trial. A total of 100 patients scheduled for, or clinically admitted after a major cardiac intervention will be recruited at Maxima Medical Center in both Eindhoven and Veldhoven. All participants are requested to provide written informed consent before study entry. Demographic and other patient-relevant data are collected at baseline: one week before intervention, or as soon as possible after intervention if it is not possible to include the patient before intervention. Periodic data are collected at baseline, one week, three months, six months, nine months and twelve months after intervention. Continuous lifestyle data are collected 7 days per month for one year after the intervention. The protocol for this study was approved by the Institutional Review Board of Maxima Medical Centre Veldhoven in the Netherlands. The trial is registered at the Netherlands Trial Registry (NTR: NL9861). An overview of the study design is provided in Fig. 1.

### Study population

Patients scheduled for or recently having undergone coronary artery bypass surgery (CABG), a fractional flow reserve test (FFR) and/or a percutaneous coronary intervention (PCI), an electrophysiology study (EP) and/or radiofrequency catheter ablation (RFCA), a transcatheter aortic valve implantation (TAVI), and/or valve surgery will be considered for participation. We will include a total of 100 patients. Patients included before their intervention will be monitored one week extra (the week prior to the intervention). A complete list of inclusion and exclusion criteria is provided in Table 1.

**Table 1:** Inclusion and exclusion criteria for Care-On

***Inclusion criteria***	
***i***	Patients selected for or that underwent coronary artery bypass surgery (CABG), a fractional flow reserve test (FFR) and/or a percutaneous coronary intervention (PCI), an electrophysiological test (EFO) and/or radiofrequency catheter ablation (RFCA), a transcatheter aortic valve implantation (TAVI), and/or valve surgery
***ii***	Age ≥ 18 years
***iii***	Able to speak and read the Dutch language
***iv***	Willing and able to provide informed consent
***Exclusion criteria***	
***i***	No internet connection at home
***ii***	Not in possession of a computer or tablet; and mobile phone
***iii***	Not able or willing to wear activity tracker on a daily basis (for example due to work related obligations)
***iv***	Major planned (cardiac) surgery in the upcoming 3 months
***v***	Life expectancy < 1 year (e.g., severe renal disease, metastatic cancer)
***vi***	Physical impairments interfering with the lifestyle monitoring system, including not able to perform daily physical activities due to orthopedic or neurological disease, bed/chair ridden patients, visual impairments/blindness, severe cognitive disability
***vii***	Presence of wounds, injuries or infectious diseases on the skin where the wrist-wearable device(s) will be placed
***viii***	Mentally incompetent

After signing informed consent patients will receive instructions to use the lifestyle monitoring platform as part of the intake procedure with the researcher and nurse specialist. During this consultation patients will be instructed to use the platform on their personal computer and personal mobile phone. Patients will also receive a health watch, the Philips Health Band (PHB) (Health Band, Koninklijke Philips N.V. (KPNV), Amsterdam, The Netherlands).

### Design of the lifestyle monitoring system

We aim to develop a platform that can adequately monitor lifestyle behaviors of cardiac patients in an integrated and holistic manner. For each lifestyle domain (physical activity, sleep, nutrition and stress) we consulted domain experts to determine which method to use, what data to collect and in what frequency we would be able to gain sufficient insight in the patient's lifestyle behavior. Secondly, a systematic review is being conducted [15] on validated self-assessment tools for cardiovascular risk behavior. Based on these sources, we chose data collection methods and set a data collection scheme. We created a Minimum Viable Product (MVP) of the lifestyle monitoring system and tested the platform with seven researchers, two dieticians with the aim to go through several quick design cycles (one week uses). We asked one patient to test the system for 15 weeks (from October 2021 to February 2022) and to provide feedback over time. These combined insights were used to mature the lifestyle monitoring system to a study ready state.

### The lifestyle monitoring plan

The design process resulted in a combination of evidence-based monitoring methods and frequencies to monitor lifestyle efficiently, accurately, and with focus on the users’ needs.

#### Continuous lifestyle monitoring

Physical activity, daily nutrition intake, stress levels and sleep quality will be monitored continuously via the Care-On lifestyle monitoring system both objectively and subjective. The methods are displayed in Table 2.

**Table 2:** Continuous lifestyle monitoring plan

**Lifestyle behaviour**	**Method**	**Subjective/objective**	**Minimum frequency per month/four weeks**
***Objective continuous lifestyle monitoring***			
Daily physical activity	24/7 activity monitoring via health watch (PHB)	Objective	Habitual physical activity 3–5 days of monitoring; sedentary: 5 days of monitoring [16]
***Subjective continuous lifestyle monitoring***			
Daily dietary habits and drinking behaviour	Self-report via chatbot based on 24 h food diary	Subjective	2–3 days of food records (preferably at least one weekday and one weekend day) [17]
Daily mental stress levels	Self-report via chatbot based on PANAS-scale (as Ecological Momentary Assessment (EMA) [18])	Subjective	No specific frequency, EMA can be adjusted to the design of the study
Daily sleep habits (quantity and quality)	Self-reported via chatbot based on Consensus Sleep Diary [19]; (additionally objectively via health watch)	Subjective (and objective)	At least 7 days including a weekend day [20] (Sleep diary); (5 nights for health watch [21])

The methods were combined in one scheme with a focus on minimizing patient burden and creating regularity. Therefore, the basis of the scheme consists of seven measurement days (based on the minimum habitual sleep measurement frequency, and this includes all days of the week). These seven measurement days are divided over two weeks (for 3 or 4 days per week (2 or 3 weekdays and 1 weekend day)) per four weeks to minimize the activity of the chatbot per week and include measurement free weeks (pause weeks). The first week of reporting contains four reporting days: Monday, Wednesday, Friday and Sunday. The second week of reporting contains three reporting days: Tuesday, Thursday and Saturday. The reporting weeks will take place every other week. Thus, in one month seven days (one complete week of reporting) of measurements is collected. Patients will be asked to wear the health watch in the weeks the chatbot is active to simplify the scheme. The chatbot and health watch scheme can be found in Table 3.

**Table 3:** Continuous lifestyle monitoring scheme: “C” = day the chatbot is active. “Wear health watch” = patient is instructed to wear the health watch during the day (night is optional). In the row below the 'Week number' the days of the week are displayed from Monday (M) to Sunday (S)

Week 1							Week 2							Week 3							Week 4						
**M**	**T**	**W**	**T**	**F**	**S**	**S**	**M**	**T**	**W**	**T**	**F**	**S**	**S**	**M**	**T**	**W**	**T**	**F**	**S**	**S**	**M**	**T**	**W**	**T**	**F**	**S**	**S**
**C**		**C**		**C**		**C**									**C**		**C**		**C**								
Wear health watch							Pause							Wear health watch							Pause						

A description of the monitoring method and frequency of each lifestyle domain is described in the following sections.

##### Daily physical activity level

Daily physical activity will be measured using a health watch (the Philips Health Band (PHB)) [22]. The health watch measures: activity counts, heartrate, respiration rate, total energy expenditure, active energy expenditure, steps, activity type, amount of time the watch has been worn, resting heartrate, recovery heartrate, cardio fitness index and the amount of time a person was asleep or awake.

Patients are asked to wear the health watch for 2 weeks per month (Table 3) for at least 12 h a day, excluding periods for charging the device and periods where the device cannot be worn (such as during washing, showering and swimming).

##### Daily dietary habits and drinking habit

Eating habits and drinking habits are measured via the chatbot, for seven days per month (Table 3). Participants will be asked to complete a 24 h food diary. The chatbot will aid in filling in the food dairy by prompting the patients after breakfast, after lunch, after dinner and the following morning to fill in their nutrition intake. Patients are prompted to report on the following morning as well to capture whether the patient has eaten something after dinner and before going to bed.

Daily nutrition intake is based on the Dutch Dietary Guideline of 2015 [23] and the Eetscore Food Frequency Questionnaire (FFQ) [24], and assessed through the following categories: vegetables; fruit; legumes; nuts and peanuts; dairy, cheese and cream; bread, cereal products and potatoes; meat, meat substitutes and egg; fish and shellfish; butter, fat and oil; drinks; salt; soup; sugar and confectionery; cake and pastry, savoury snacks and fast food.

##### Daily mental stress levels

Daily self-reported mental stress levels are measured via the chatbot using Ecological Momentary Assessment (EMA) with questions derived on the Positive and Negative Affect Schedule (PANAS) [25]. The chatbot will ask participants to indicate their feelings 3 times per day (‘How did you feel over the past 10 min including now?’): at wake up, after lunch and after dinner (together with the nutrition notification). Participants are asked to what extent (on a 5-point Likert-scale: 0 = ‘very slightly or not at all’, 1 = ‘a little’, 2 = ‘moderately’, 3 = ‘quite a bit’ and 4 = ‘extremely’) they experience the following states: stressed, happy, irritated, anxious, active, whether they have control over what they do, feeling physically well (and if not, what is bothering them), a personalized stress symptom (the patient will be asked to provide this personalized stress symptom during first time use of the chatbot).

##### Daily sleep habits

Daily self-reported sleep quality and quantity is measured via the chatbot based on the Consensus Sleep Diary [19]. 5 items will be asked via the chatbot in the morning when the participant wakes up, with the following outcomes: usual bedtime (time), usual getting up time (time), hours of sleep per night (hours), perceived quality of sleep (Likert-scale), perceived feeling of restfulness (Likert-scale). Secondly, the PHB will objectively measure sleep quantity and quality.

#### Periodic lifestyle assessment

Patients will be asked to fill in a set of questionnaires (quarterly assessment) at baseline (t0) (one week before intervention), one week after discharge (t1) and at 3 months (t2), 6 month (t3), 9 months (t4) and 12 months after discharge (t5). The results of  the questionnaires related to the patients lifestyle (as described in Table 4) are visualized with a score in the lifestyle monitoring system.

**Table 4:** Periodic lifestyle assessment overview

*Periodic lifestyle assessments (quarterly)*		
**Lifestyle parameter**	**Questionnaire**	**Objective**
Perceived physical fitness	FitMáx	The FitMáx [26] used to assess perceived physical fitness as an addition to the daily physical activity data measured by the PHB
Diet quality	NutriMáx (custom FFQ)	A customized healthy diet assessment questionnaire, the NutriMáx (a custom questionnaire based on Dutch Dietary Guideline [23]) is used to assess diet quality and nutrition behavior as an addition to the daily eating habits
Smoking status	Custom questionnaire	The participants will be asked to indicate their smoking status and average amount of smoking (cigarettes per day)
Perceived mental stress	Perceived Stress Scale (PSS-10)	The PSS-10 [27] is used to assess perceived mental stress as an addition to the daily stress level measurements
Perceived sleep quality	Pittsburgh Sleep Quality Index (PSQI)	The PSQI [28] is used to assess perceived sleep quality

### The lifestyle monitoring system for patients

Patients will receive an account for the Care-On lifestyle monitoring system for administering daily health data. The patients will be asked to install the application of the platform (MijnFlowCoach [29]) on their mobile phone or tablet and get additional instructions to access the platform on their desktop.

The Care-On platform for lifestyle monitoring contains five main features:

- **Health watch (PHB) integration:** Upload and review daily physical activity data collected via an integrated wrist-worn wearable device.
- **Chatbot module:** The chatbot module will prompt the patients to self-report on their lifestyle behaviour regarding mental stress, nutrition intake and sleep habits.
  - Measuring daily dietary habits and drinking habit
  - Measuring daily mental stress
  - Measuring daily sleeping habits

- **A personal dashboard:** A personal dashboard will give feedback on the patients’ adherence to self-report their lifestyle behaviour and provide an overview of the self-reported lifestyle data. Secondly, every quarter patients will be asked to fill in more extensive questionnaires on lifestyle parameters as an addition to the daily chatbot and smartwatch integration measurements via the personal dashboard.
- **A goal tracking module:** The patient has the option to set and monitor monthly goals with the goals tracking module.

**Fig. 2:**
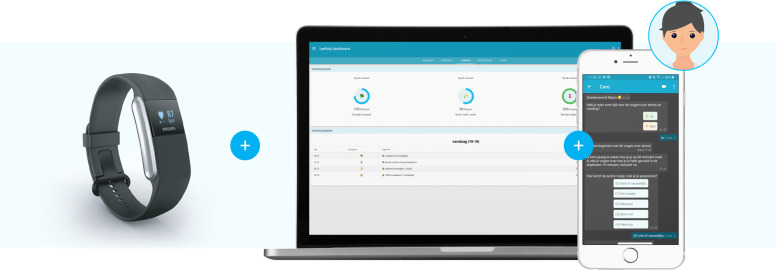
Care-On lifestyle monitoring system with integrated health watch and lifestyle chatbot

An impression is shown in Fig. 2.

#### Health watch

The PHB will be integrated in the platform and data visualizations of the data collected with the watch will be visualized on a personal dashboard. The patient will not be treated or coached based on the parameters measured by the devices as caretakers do not have access to these data. The patient will wear the device during a period of one week before intervention (if possible) and one year after intervention. After this period the patient will hand in the devices.

Patients are instructed to wear the PHB at least 12 h per day. The PHB will be paired with the mobile phone of the patient via the ‘Philips Gezondheid band’ application [30] which will be installed on the patient's phone at first visit. The patient is instructed to open the application to synchronize the data of the watch with the Philips-application once every two days. The application will provide the patients with data visualizations of the data collected by the PHB. The patients may wear the PHB continuously (also in weeks of no chatbot measurements) and during the night by choice.

#### Chatbot

A conversational agent (chatbot), called ‘Caro’, is integrated in the Care-On platform. Caro is represented by a female avatar and is programmed to ask the patients questions to monitor the lifestyle parameters sleep, stress, and nutrition intake.

During a measurement day Caro starts a chat session five times per measurement day: in the morning (sleep and stress), after breakfast (nutrition), after lunch (stress and nutrition) and after dinner (stress and nutrition) and the morning after (nutrition). During onboarding Caro will ask patients to fill in their daily schedule to personalize the chat timers.

If a patient is not able to answer a question directly, Caro will ask again after 30 min. The patient has until the next chat session to answer the questions. If a patient has answered all questions of the chat session the measurement is completed and counted for compliance.

**Fig. 3:**
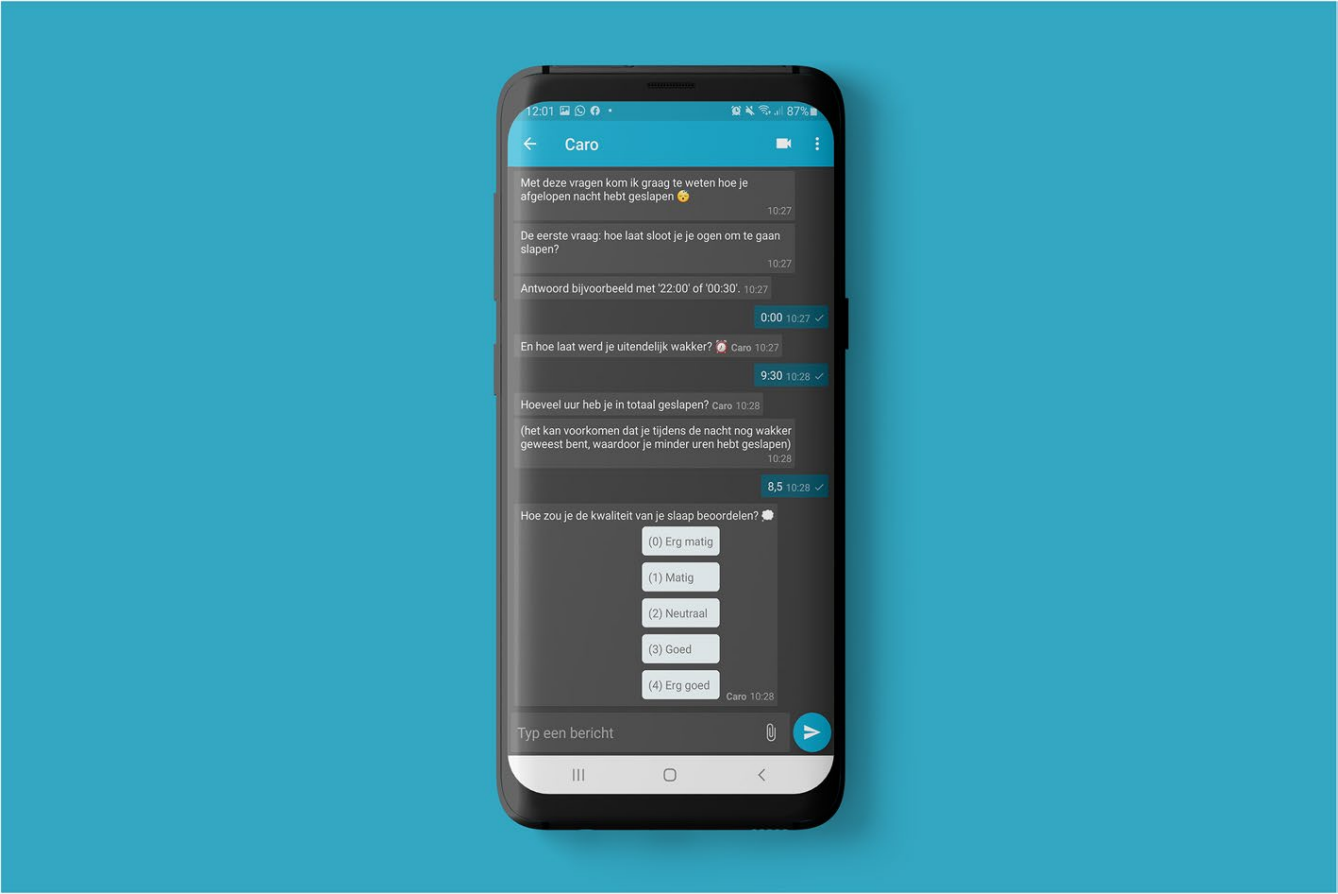
Example of chatbot (Caro) questions regarding sleep quantity and quality

The chatbot is available via the platform on desktop and via the ‘MijnFlowCoach’-application [29] which is installed on the patient's phone during onboarding. An impression is shown in Fig. 3.

#### Personal dashboard

**Fig. 4:**
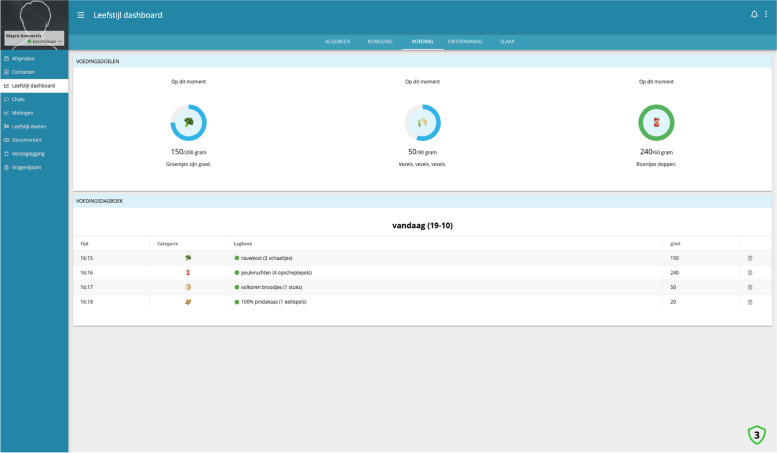
Personal dashboard: nutrition overview including visualized lifestyle goals

The data acquired via the PHB, the chatbot and the periodic lifestyle assessments are visualized and accessible for the patient in a dashboard in the lifestyle monitoring system. The data visualized on the dashboard is focused on the adherence to delivering self-reported data to the system and the content of their delivered data. An example is shown in Fig. 4.

The periodic lifestyle assessments are also available via the dashboard. After having completed the periodic lifestyle assessment basic advice on the results is shown in the dashboard.

#### Goal tracking module

The patient has the option to set and monitor monthly goals with the goal tracking module. This module is optional. Default goals for dietary intake and physical activity will be provided based on the Dutch Dietary Guideline of 2015 and Dutch Physical Activity Guideline of 2017 [31]. The default goals for sleep and stress are based on the advice given by the Dutch General Practitioners Associations (in Dutch ‘Nederlands Huisartsen Genootschap (NHG)’) on the ‘Thuisarts’-website [32, 33]. The patient has the option to alter the default goals to fit their personal situation.

#### Quarterly consults by research nurse and final evaluation

Every quarter the research nurse will contact the patient via phone consultation to ask whether the patient has problems using the system. Secondly, the research nurse will ask for brief feedback on the use of the system. Thirdly, the research nurse will remind the patient to fill in the quarterly assessment when the patient has not completed it yet. At last, the research nurse will ask the patient to report medical events.

Within two weeks after the trial period has ended the SUS will be administered again and a semi-structured interview will be held with the participant to discuss their experience with the lifestyle monitoring system. During the semi-structure interview the following topics will be discussed: their quality of life after intervention, their experience with cardiac rehabilitation and/or cardiac care after intervention, their experience with the Care-On study in general, and their perceived usability of the different functionalities of the lifestyle monitoring system.

### Outcome measures

#### Primary endpoint

The primary outcome is adherence providing self-tracking data, which is calculated as the total number of participants that completed the study at one year follow-up divided by the number who started using the system at baseline. Secondly, the general adherence rate and individual adherence rates of the chatbot, the quarterly assessments and wearing the health watch are compared. Adherence classifications and a dropout threshold were set up to adequately categorize patient’s adherence (see next section ‘Adherence classification and dropout threshold’). Other measures of adherence will also be explored (i.e., adherence rates per quarter, the success rate (percentage of the tasks that the patients complete correctly)).

##### Adherence classification and dropout threshold

Classifications for low, moderate and high adherence are set up to monitor patient’s compliance with providing lifestyle data. The health watch adherence rate, chatbot adherence rate and quarterly assessment completion rates make one-third of the general adherence rate:

$$general\,adherence\,rate\,=\,\left(adherence\,rate\,PHB\,+\,adherence\,rate\,chatbot\,+\,quarterly\,assessment\,completion\,rate\right)/3$$

The chatbot compliance rate is based on the minimum number of days of data collection necessary to gain insight in habitual intake of commonly consumed food. The minimum number of days of data to be collected to gain insight in habitual intake of commonly consumed food is 2–3 days (of food records, real-time monitoring) [17]. Thus, if a patient fills in at least 50% of the measurements, the minimum number of days is certainly met.

The health watch compliance rate is based on the minimum number of days necessary to gain insight in habitual physical activity and sedentary behavior. Previous studies [16] indicate that 3–5 days of monitoring is necessary to assess habitual PA and for sedentary behavior 5-days of monitoring will provide a reliable estimate. Patients were instructed to wear the PHB for at least 12 h a day on average during measurement weeks. Thus, if a patient wears the watch at least 50% of the prescribed time, the minimum number of days certainly is met.

The quarterly lifestyle assessment completion rate is based on the amount of completed quarterly assessments.

The adherence to providing self-tracking data is evaluated by the researchers every four weeks per patient. The total adherence score is a combined score of the chatbot adherence, activity tracker wear adherence and quarterly assessment adherence. The levels are as follows:

- Low adherence classification: The patient's adherence is below 50% measured over a 4-week interval.
- Moderate adherence classification: The patient's adherence is between 50 and 75% measured over a 4-week interval.
- High adherence: The patient’s adherence is above 75% measured over a 4-week interval.

Patients who have a general adherence rate below 50% will be contacted and stimulated to resume using the system by the research nurse once; if patients did not resume after 1 month they are classified as non-adherent.

#### Secondary endpoints

The secondary endpoints are:

- Usability (measured using the SUS, the feedback given by the patients during the phone consults with the research nurse, the quarterly questionnaires and the evaluation interview) and the success rate (percentage of tasks that the patients complete correctly)).
- Predictors of adherence to the lifestyle monitoring system (demographic and disease characteristics, quality of life, self-efficacy, depressive symptoms and anxiety, motivation, stage of change, fatigue, physical fitness, levels of metal stress, use of a goal tracking functionality, perception of system usability and prior experience with technology. Standardized questionnaires will be used for self-report measures and objective ambulatory measures from the lifestyle monitoring system for objective lifestyle measures).
- The association between lifestyle behaviour at baseline with clinical outcomes will be examined by evaluating patient clinical records (re-hospitalizations related to cardiac disease) and standardized self-report data (quality of life).

The data will be analyzed using multiple regression analysis. An overview of the questionnaires to be complete by the patients per timepoint for the secondary endpoints is shown in Table 5.

**Table 5:** Overview of the assessments of the secondary endpoints of the Care-On study

		**STUDY PERIOD**						
		Baseline	Rehabilitation					Evaluation
***TIMEPOINT***		***-t1***	***t1***^***2***^	***t2***	***t3***	***t4***	***t5***	***t6***
*Predictors of adherence*								
Health-related Quality of Life	Short Form Health Survey (SF-12) v1 [34]	x	x	x	x	x	x	
Self-efficacy	General Self-Efficacy Scale (GSES) [35]	x						
Depressive symptoms and anxiety	Hospital Anxiety and Depression Scale (HADS) [36]	x						
Motivation and Stage of Change	Readiness-to-Change Lifestyle Questionnaire and Confidence-to-Change Lifestyle Questionnaire [37]	x						
Fatigue	Fatigue Assessment Scale (FAS) [38]	x						
Physical fitness^1^	FitMáx [26]	x	x	x	x	x	x	
Levels of mental stress^1^	PSS-10 [27]	x	x	x	x	x	x	
System usability	SUS [39]	x						x
Mobile Device Proficiency	Mobile Device Proficiency Questionnaire (MDPQ)-16 [40]	x						
*Clinical data*								
Medication adherence	General Adherence Scale (GAS) [41]	x	x	x	x	x	x	
*Usability*								
Satisfaction and usability	Custom questionnaire based on the customer satisfaction score (CSAT) [42] and single ease questions (SEQ) [43]		x	x	x	x	x	
Self-report chatbot and activity tracker usage	Custom questionnaire based on GAS		x	x	x	x	x	

^1^These questionnaires are also part of the periodic lifestyle assessment

^2^The questionnaires of 't1' will not be asked when a patient is included after intervention

### Sample size calculation and statistical analysis

The sample size is based on the primary objective adherence. Anticipating a dropout rate of 50% at 1 year follow up, 100 patients are needed to estimate this adherence rate with a precision of ± 10% (95% CI = [0.398, 0.601]).

Descriptive statistics will be used to report demographics and baseline characteristics. The primary outcome (i.e., ‘adherence’ based on continued use status at 1-year follow-up) will be analysed as a dichotomous outcome measure. Predictors of adherence status and which factors predict re-hospitalizations related to cardiac disease (i.e., the secondary aims) will be investigated using multiple logistic regression analyses. Predictors continuous outcome measures (e.g., QoL) will be examined using linear regression models. Analysis will be carried out in the statistical software package SPSS (version 24, SPSS Inc.).

### Trial status

The Institutional Review Board of the hospital has approved the study protocol and its amendments prior to the start of the study. The inclusion of the patients started in November 2021 and is expected to be completed in June 2023. The expected study end date is one year (June 2024) later as the last included patient has finished their study year.

## Discussion

The Care-On trial is one of the first trials to evaluate long term adherence to monitoring multiple lifestyle behaviour domains with a monitoring system after major cardiac events. We postulate that a system that aids patients in monitoring their lifestyle will enable better self-management and improve self-motivation [14], with subsequent positive effects on the lifestyle factors themselves and, eventually long-term health outcomes. Adherence to monitoring devices that continuously provide self-tracking data is key and therefore the primary outcome of this study. Furthermore, we investigate the determinants of adherence to and usability of the system to further improve and personalize the lifestyle monitoring system to be easily integrated in the cardiac rehabilitation (CR) programs and to, consecutively, enhance adherence and eventually (lifestyle) behavior change.

For cardiac patients, CR programs are crucial for secondary prevention of cardiovascular events and optimization of risk factors and lifestyle behavior. Despite the undisputed advantages of participation in CR, too few patients participate in CR programs and adherence to maintaining a healthy lifestyle after a cardiac event or intervention is poor [44]. Secondly, standard CR and tele-rehabilitation programs aim to accomplish and maintain healthy lifestyle behaviour, however we often see a relapse in lifestyle behaviour during and after CR programs [45, 46]. The shortcomings of current CR programs may at least be partly due to insufficient long-term guidance by medical professionals, insufficient focus on sustainable behavioral change and self-management, and the fact that current group-based CR programs often do not meet patients’ individual needs and competences [47]. The latter may contribute to suboptimal adherence to prescriptions of lifestyle behavior (e.g. daily physical activity, dietary advice) and advices on self-monitoring, both in conventional CR programs and cardiac tele-rehabilitation programs. There is a clear need for long term strategies for sustainable behaviour change to achieve long term results.

Increased adherence to self-monitoring of lifestyle behavior can improve behavioral change by incorporating self-monitoring into patients’ daily routines, thus stimulating the development of self-management skills [48–50], even beyond phase II CR programs. Also, these data enable health care professionals to provide more suitable and sustainable advices and guidance. We envision that these insights will contribute to identify profiles that will help in optimizing personalized decision making. Also, insights in patients lifestyle data will add in achieving more long-lasting improvement in lifestyle as personalized regimes can be applied (e.g., pre and/or post-intervention rehabilitation, lifestyle management). Furthermore, continuous monitoring of lifestyle behaviour may enable early detection and treatment of clinical deterioration which will have a positive impact on the disease course (e.g., prevention of heart failure).

Research on the adherence to continuous lifestyle monitoring technologies for a longer period of time is scarce. In fact, previous research showing high levels of adherence associated with monitoring technology focused on relatively short programs (1 week to 6 months). Furthermore, these studies typically focused on monitoring only one or two lifestyle domains rather than all lifestyle domains that are part of secondary prevention cardiovascular diseases. The Care-On lifestyle system plan has been designed with a user-centered design approach to design a system suitable for long term use and monitoring of multiple lifestyle domains. Monitoring methods where chosen based on two important factors to optimize adherence: high usability in combination with low patient burden. The result was a lifestyle monitoring system with integrated chatbot and a wearable device, with a monitoring plan that is tailored to the patients’ daily routines.

## Conclusion

The Care-On study investigates a newly designed lifestyle monitoring system for cardiac patients that enables long-term continuous monitoring of multiple lifestyle domains. It will provide insight in the adherence and usability of continuous lifestyle monitoring and it will give insight into the association between lifestyle behavior and clinical outcomes and patient-relevant outcomes. These insights can enable the design of personalized cardiac interventions, which may lead to enhanced patient participation and improved long-term adherence in cardiac rehabilitation, resulting in improved patient-relevant outcomes and reduce healthcare costs.

## Data Availability

Not applicable.

## Abbreviations

CR: Cardiac rehabilitation
PA: Physical activity
CVD: Cardiovascular disease
CAD: Coronary artery disease
AF: Atrial fibrillation
CABG: Coronary artery bypass grafting
RFCA: Radiofrequency catheter ablation
EP: Electrophysiology study
TAVI: Transcatheter aortic valve implantation
FFR: Fractional flow reserve test
PCI: Percutaneous coronary intervention
NTR: Netherlands trial registry
PHB: Philips Health Band
KPNV: Koninklijke Philips N.V
MVP: Minimum viable product
FFQ: Food frequency questionnaire
PANAS: Positive and Negative Affect Schedule
EMA: Ecological momentary assessment
PSS: Perceived Stress Scale
PSQI: Pittsburgh Sleep Quality Index
iOS: IPhone Operating System
NHG: Nederlands Huisartsen Genootschap
SUS: System Usability Scale
QoL: Quality of Life
SF-12: Short Form Health Survey
GSES: General Self-Efficacy Scale
HADS: Hospital Anxiety and Depression Scale
FAS: Fatigue Assessment Scale
MDPQ: Mobile Device Proficiency Questionnaire
GAS: General Adherence Scale
CI: Confidence interval
SPSS: Statistical package for the social sciences
CSAT: Consumer satisfaction score
SEQ: Single ease question
